# β-Adrenoreceptor Stimulation Mediates Reconsolidation of Social Reward-Related Memories

**DOI:** 10.1371/journal.pone.0039639

**Published:** 2012-06-20

**Authors:** E. J. Marijke Achterberg, Viviana Trezza, Louk J. M. J. Vanderschuren

**Affiliations:** 1 Division of Behavioural Neuroscience, Department of Animals in Science and Society, Faculty of Veterinary Medicine, Utrecht University, Utrecht, The Netherlands; 2 Department of Neuroscience and Pharmacology, Rudolf Magnus Institute of Neuroscience, University Medical Center Utrecht, Utrecht, The Netherlands; 3 Department of Biology, University “Roma Tre”, Rome, Italy; Radboud University, Netherlands

## Abstract

**Background:**

In recent years, the notion that consolidated memories become transiently unstable after retrieval and require reconsolidation to persist for later use has received strong experimental support. To date, the majority of studies on reconsolidation have focused on memories of negative emotions, while the dynamics of positive memories have been less well studied. Social play, the most characteristic social behavior displayed by young mammals, is important for social and cognitive development. It has strong rewarding properties, illustrated by the fact that it can induce conditioned place preference (CPP). In order to understand the dynamics of positive social memories, we evaluated the effect of propranolol, a β-adrenoreceptor antagonist known to influence a variety of memory processes, on acquisition, consolidation, retrieval and reconsolidation of social play-induced CPP in adolescent rats.

**Methodology/Principal Findings:**

Systemic treatment with propranolol, immediately before or after a CPP test (i.e. retrieval session), attenuated CPP 24 h later. Following extinction, CPP could be reinstated in saline- but not in propranolol-treated rats, indicating that propranolol treatment had persistently disrupted the CPP memory trace. Propranolol did not affect social play-induced CPP in the absence of memory retrieval or when administered 1 h or 6 h after retrieval. Furthermore, propranolol did not affect acquisition, consolidation or retrieval of social play-induced CPP.

**Conclusions/Significance:**

We conclude that β-adrenergic neurotransmission selectively mediates the reconsolidation, but not other processes involved in the storage and stability of social reward-related memories in adolescent rats. These data support the notion that consolidation and reconsolidation of social reward-related memories in adolescent rats rely on distinct neural mechanisms.

## Introduction

A newly acquired memory is initially unstable and prone to both facilitation and impairment. Memory consolidation progressively stabilizes the memory, making it resistant to interference [1]. However, retrieval of a consolidated memory has been found to cause the memory to become unstable, in the sense that it is again vulnerable to interference. Reconsolidation is the process by which a retrieved memory is stabilized again [2,3,4;5,6,7]. The function of memory reconsolidation is a topic of debate. Recent studies propose that reconsolidation is a process for maintaining and strengthening memory to prevent forgetting [8] or to incorporate new information into the reactivated memory-trace [7]. Reconsolidation is usually studied using aversive memories. There is also a substantial literature about the reconsolidation of food and drug memories, but reconsolidation of memories of physiologically relevant natural rewards such as social stimuli, has received little attention [9].

Social play is the most characteristic social behavior in adolescent mammals, which serves to facilitate social, physical and cognitive development [10]–[13] Social play is highly rewarding for adolescent rats [11], [14], [15] as exemplified by its capacity to induce conditioned place preference (CPP) [16–20]. Because place conditioning relies on an associative mechanism, it can be used to study the dynamics of emotionally charged memories [21], [22].

The β-adrenergic receptor has been implicated in memory reconsolidation for aversive as well as for pleasurable stimuli and events. For example, systemic administration of β-adrenergic antagonists such as propranolol (PROP) induces a memory impairment in rats in tasks such as fear conditioning [23], conditioned stimulus-induced cocaine or sucrose seeking [24], [25], and drug-induced CPP [21], [22], [26]. PROP has also been shown to disrupt reconsolidation of fear memory in humans [27].

In the present study, we investigated whether retrieved social reward-related memories in a social play-induced CPP paradigm could be disrupted by administration of PROP in adolescent rats. We hypothesized that if social reward-related memories reconsolidate following memory retrieval, PROP would attenuate preference for a social play-paired environment by disrupting the memory trace. This would prevent reinstatement of CPP following extinction and retraining. We also investigated the period of instability of the social play memory after retrieval (reconsolidation-window). Furthermore, since β-adrenergic signaling has also been implicated in other aspects of learning and memory [1], [28], we also tested whether PROP affected the acquisition, consolidation and retrieval of social play-induced CPP.

## Results

### 1. Effects of acute post-retrieval PROP on social play-induced CPP

**Figure 1:**
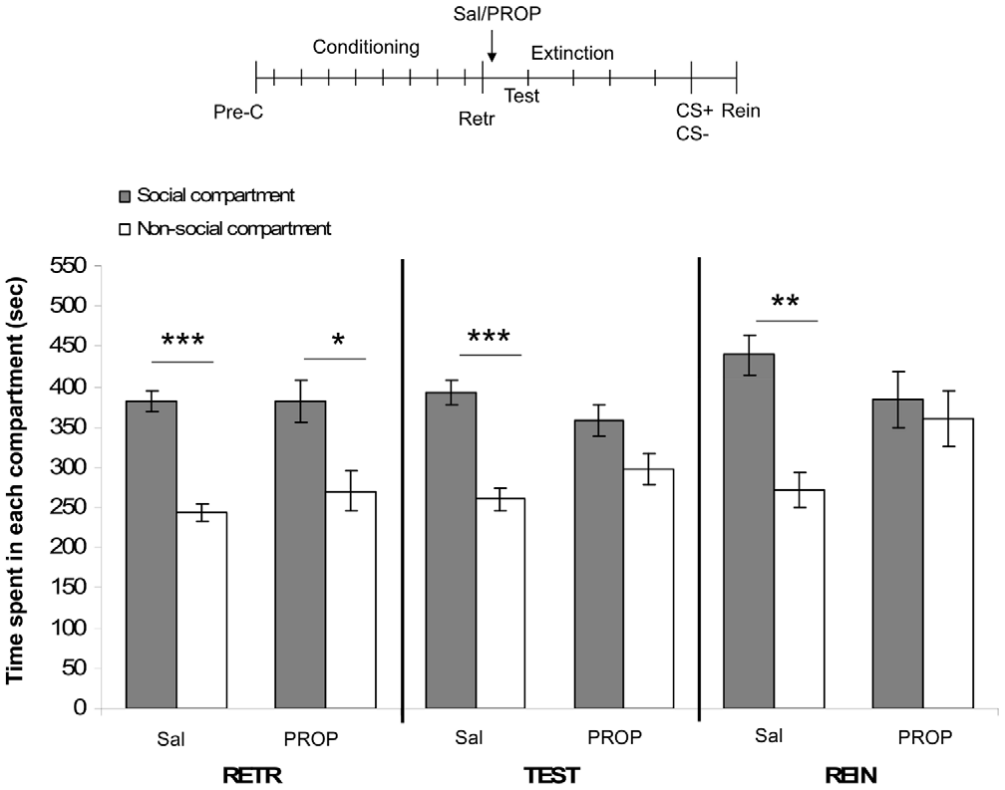
Effects of acute post-retrieval PROP on social play-induced CPP. The experimental protocol is depicted above the graph (Pre-C: pre-conditioning test, CS+: conditioning session with a play-partner, CS-: conditioning session alone). Data represent the mean time (sec ± SEM) spent in the social compartment (grey bars) and the non-social compartment (white bars) during 15 min retrieval- (RETR), test- (TEST) and reinstatement- (REIN) sessions. Saline-treated animals (2 ml/kg, *i.p.*, n = 18), PROP-treated animals (10 mg/kg, *i.p.*, n = 8). Post-hoc Student's paired t-tests for difference in time spent in the social- and non-social compartment *p<0.05, **p<0.01, ***p<0.001.

The mixed-model ANOVA revealed an effect of compartment (F_(1,50)_ = 45.78, p<0.01), test-day (F_(2,100)_ = 5.88, p<0.01) and a compartment per treatment interaction (F_(1,50)_ = 6.65, p<0.05). No other main or interaction effects were found. Post-hoc tests revealed that the ‘to be’ saline-treated animals, and the ‘to be’ PROP-treated animals showed a significant preference for the social-play paired compartment on day 10 (RETR: PROP-treated rats: n = 8, t = 2.36, p = 0.05; saline-treated rats: n = 18, t = 7.35, p<0.001; Figure 1). Twenty-four hours later (TEST, Figure 1), saline-treated animals still showed a preference for the social play-paired compartment (t = 5.18, p<0.001), whereas PROP-treated animals did not (t = 1.72, p = 0.13). Following the reconditioning session, saline-treated animals showed reinstatement of social-play induced CPP (REIN: t = 3.69, p<0.01), while PROP-treated rats did not (REIN: t = 0.40, p = 0.70; Figure 1). These findings indicate that PROP treatment interferes with memory reconsolidation immediately following retrieval of the social reward memory.

### 2. Effects of delayed post-retrieval PROP on reconsolidation of social play-induced CPP

**Figure 2:**
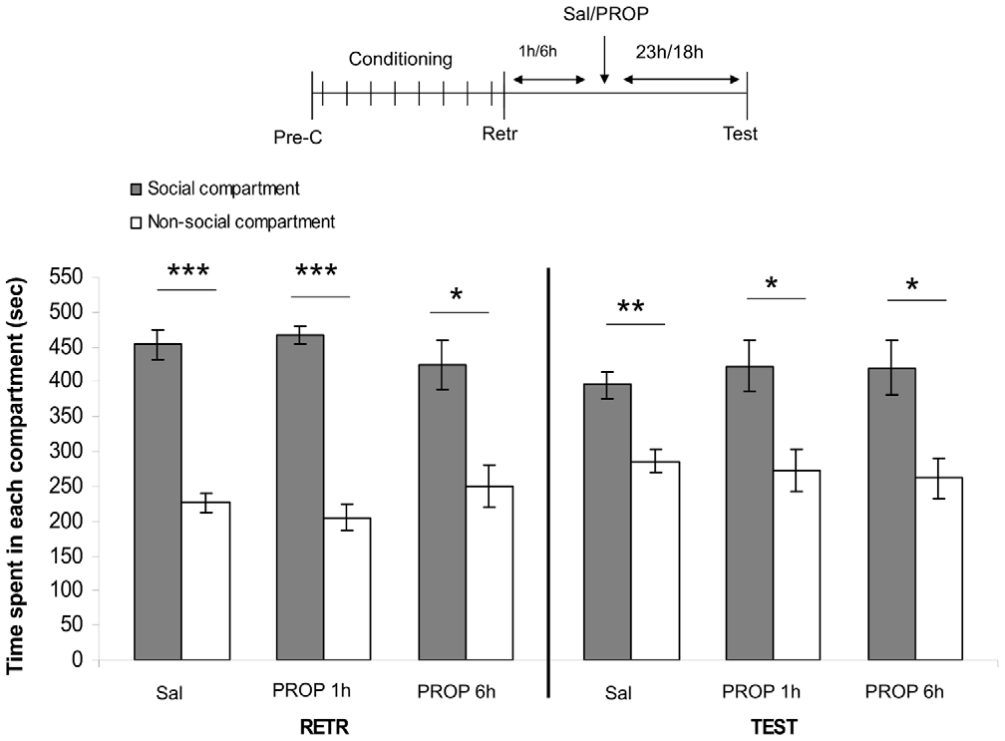
Effects of 1h and 6h delayed post-retrieval PROP on reconsolidation of social play-induced CPP. The experimental protocol is depicted above the graph (Pre-C: pre-conditioning test, CS+: conditioning session with a play-partner, CS-: conditioning session alone). Data represent the mean time (sec ± SEM) spent in the social compartment (grey bars) and the non-social compartment (white bars) during 15 min retrieval- (RETR) and test- (TEST) sessions. Saline-treated animals (2 ml/kg, *i.p*., n = 17), 1 h delayed PROP-treated animals (10 mg/kg, *i.p*., n = 13), 6 h delayed PROP-treated animals (10 mg/kg, *i.p.,* n = 10). Post-hoc Student's paired t-tests for difference in time spent in the social- and non-social compartment *p<0.05, **p<0.01, ***p<0.001.

The mixed-model ANOVA revealed an effect of compartment (F_(1,74)_ = 150.71, p<0.05). No other main or interaction effects were found. Post-hoc tests revealed that all three groups showed a significant preference for the social-paired compartment (RETR: saline-treated rats: n = 17, t = 7.09, p<0.001; 1 h delayed PROP-treated rats: n = 13, t = 9.89, p<0.001; 6 h delayed PROP-treated rats: n = 10, t = 2.82, p<0.05; Figure 2). The next day, all groups continued to show a significant preference for the social-paired compartment (TEST: saline-treated rats: t = 3.30, p<0.01; 1 h delayed PROP-treated rats: t = 2.29, p<0.05; 6 h delayed PROP-treated rats: t = 2.49, p<0.05). These data suggest that β-adrenoceptor-dependent reconsolidation of social reward-related memories takes place within 1 h after memory retrieval.

### 3. Effects of PROP on social play-induced CPP in the absence of memory retrieval

**Figure 3:**
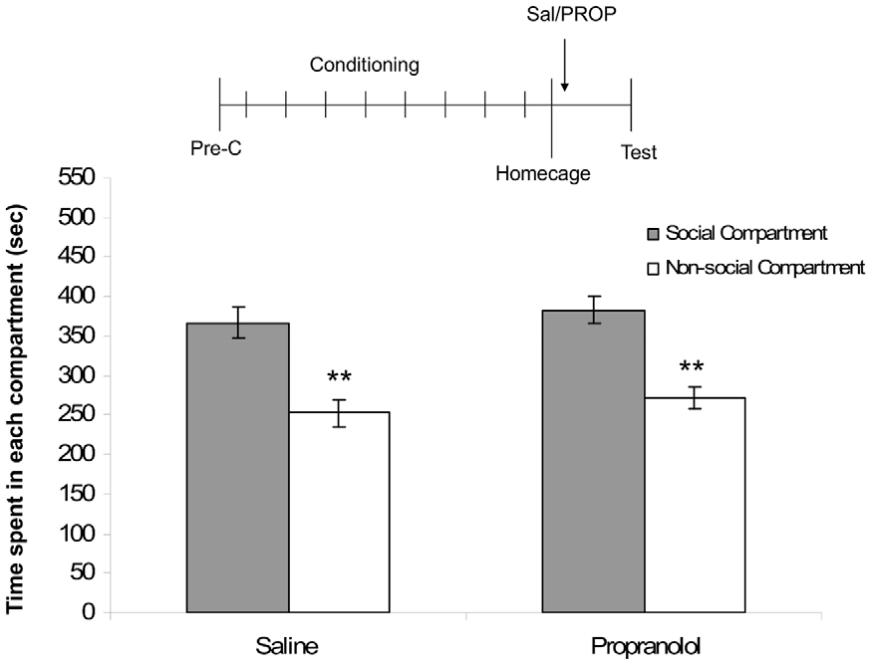
Effects of PROP on social play-induced CPP in the absence of memory-retrieval. The experimental protocol is depicted above the graph (Pre-C: pre-conditioning test). Data represent the mean time (sec ± SEM) spent in the social compartment (grey bars) and the non-social compartment (white bars) during a 15 min test session. Saline-treated animals (2 ml/kg, *i.p.*, n = 16), PROP-treated animals (10 mg/kg, *i.p.*, n = 16). Post-hoc Student's paired t-tests for difference in time spent in the social- and non-social compartment **p<0.01.

A two-way ANOVA revealed an effect of compartment (F_(1,60)_ = 44.74, p<0.05). No other main or interaction effects were found. Post-hoc tests showed that twenty-four hours after PROP or saline administration in the home-cage, animals showed a significant preference for the social-paired compartment (TEST: PROP-treated animals: n = 16, t = 3.36, p<0.01; saline-treated animals: n = 16, t = 4.03, p<0.01; Figure 3). These results indicate that memory retrieval is required for PROP to affect reconsolidation of social reward-related memories.

### 4. Effects of PROP on retrieval of social play-induced CPP

**Figure 4:**
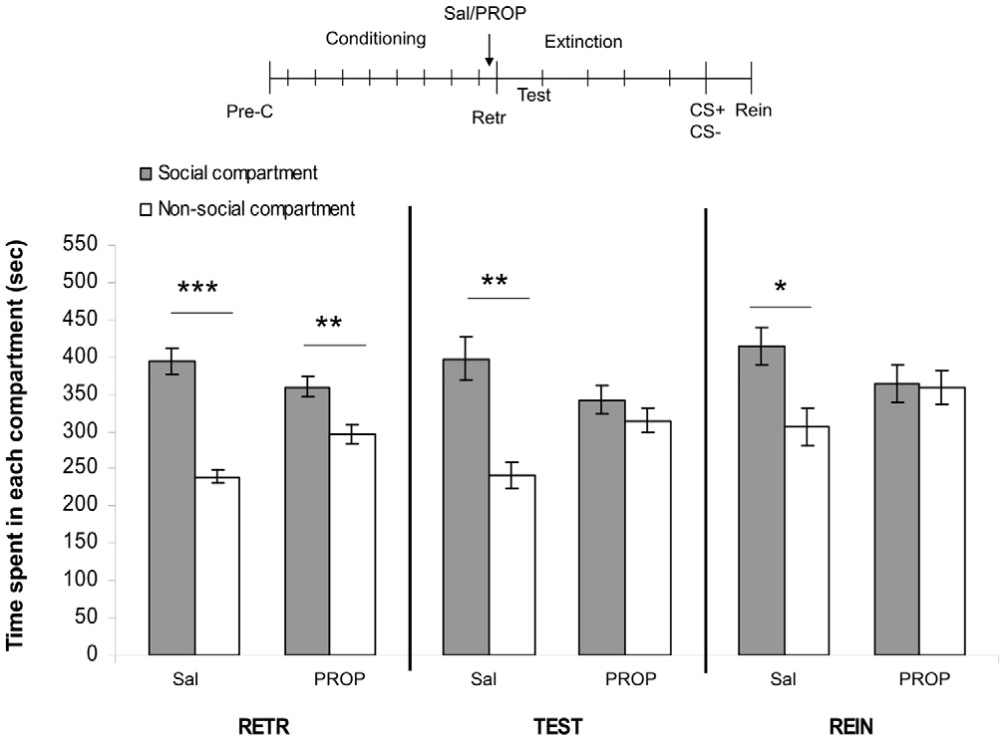
Effects of PROP on memory-retrieval of social play-induced CPP. The experimental protocol is depicted above the graph (Pre-C: pre-conditioning test, CS+: conditioning session with a play-partner, CS-: conditioning session alone). Data represent the mean time (sec ± SEM) spent in the social compartment (grey bars) and the non-social compartment (white bars) during 15 min retrieval- (RETR), test-(TEST) and reinstatement- (REIN) sessions. Saline-treated animals (2 ml/kg, *i.p*., n = 22), PROP-treated animals (10 mg/kg, *i.p.,* n = 15). Post-hoc Student's paired t-tests for difference in time spent in the social- and non-social compartment *p<0.05, **p<0.01, ***p<0.001.

The mixed model ANOVA revealed an effect of compartment (F_(1,70)_ = 34.09, p<0.05), test-day (F_(1,140)_ = 6.01, p<0.05) and a compartment per treatment interaction (F_(1,70)_ = 13.24, p<0.05). No other main or interaction effects were found. Post-hoc tests revealed that both the saline- and PROP-treated animals showed a significant preference for the social-paired compartment at retrieval (RETR: saline-treated animals: n = 15, t = 7.09, p<0.001; PROP-treated animals: n = 22, t = 2.70, p = 0.01; Figure 4). These results suggest that PROP does not affect retrieval of social reward-related memories. Twenty-four hours later, saline-treated animals continued to show a significant preference for the social-paired compartment (TEST: t = 3.61, p<0.01), while PROP-treated animals no longer showed CPP (TEST: t = 0.86, p = 0.40). After extinction and reconditioning, animals were tested for reinstatement. Saline-treated animals showed significant reinstatement of CPP whereas PROP-treated animals did not reinstate their preference (REIN: saline-treated animals: t = 2.46, p<0.05; PROP-treated animals: t = 0.11, p = 0.92). These results suggest that instead of retrieval, reconsolidation is affected by PROP, consistent with the results of experiment 1.

### 5. Effects of PROP on acquisition and consolidation of social play-induced CPP

**Figure 5:**
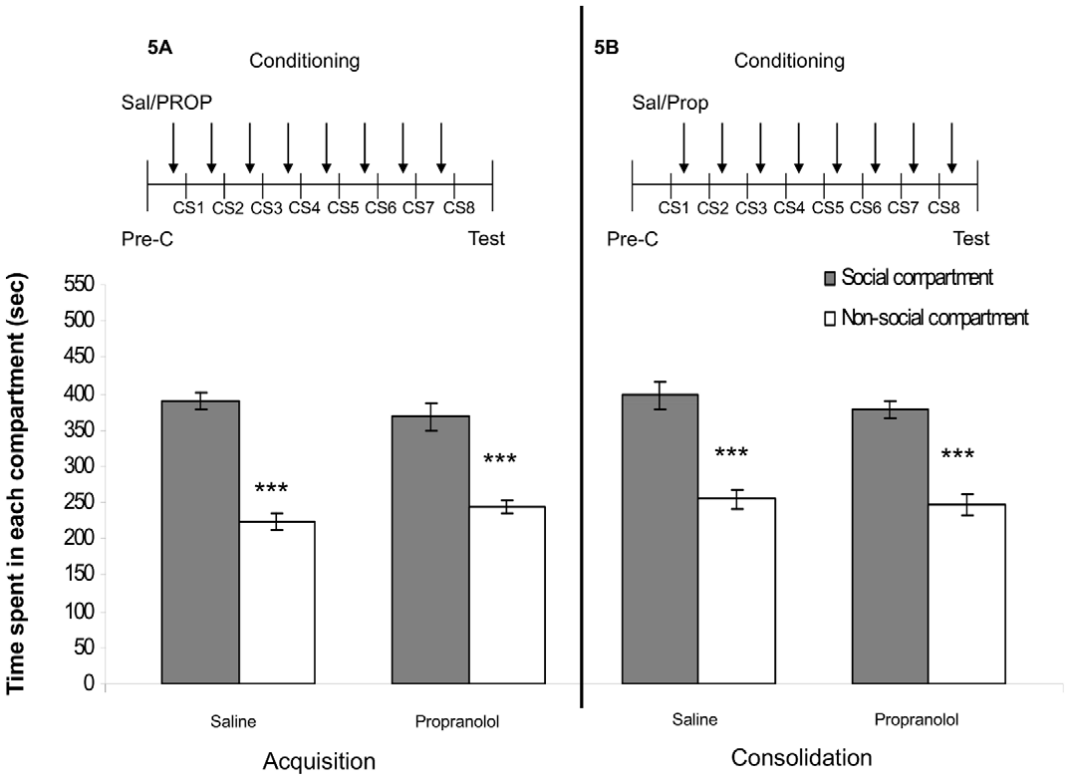
Effects of PROP on acquisition (panel A) and consolidation (panel B) of social play-induced CPP. The experimental protocol is depicted above the graph (Pre-C: pre-conditioning test, CS: daily conditioning sessions, consisting of one session with and one session without a play-partner present). PROP was administered either 30 min before (acquisition) or immediately after (consolidation) each conditioning session. Data represent the mean time (sec ± SEM) spent in the social compartment (grey bars) and the non-social compartment (white bars) during a 15 min retrieval-session. Saline-treated animals (2 ml/kg, *i.p.*, acquisition: n = 16, consolidation: n = 12), PROP-treated animals (10 mg/kg, *i.p.*, acquisition: n = 16, consolidation: n = 12). Post-hoc Student's paired t-tests for difference in time spent in the social- and non-social compartment **p<0.01, ***p<0.001.

Two-way ANOVAs revealed an effect of compartment (acquisition: F_(1,60)_ = 114.93, p<0.05; consolidation: F_(1,44)_ = 85.40, p<0.05). No other main or interaction effects were found. Post-hoc tests revealed that both the PROP- and the saline-treated animals showed a robust preference for the social-paired compartment after 8 days of conditioning (Figure 5A: acquisition: RETR: PROP-treated animals: n = 16, t = 5.24, p<0.01; saline-treated animals: n = 16, t = 7.40, p<0.01; Figure 5B: consolidation: RETR: PROP-treated animals: n = 12, t = 5.40, p<0.01; saline-treated animals: n = 12, t = 4.98, p<0.01). These results show that PROP does not affect acquisition and consolidation of social play-induced CPP.

## Discussion

In this study, we investigated the involvement of noradrenergic neurotransmission in reconsolidation of social reward-related memories in adolescent rats. Our hypothesis was that, following memory-retrieval, the β-adrenergic receptor antagonist PROP would disrupt the reconsolidation of social play-induced CPP. We show that: (1) the reconsolidation process, which has previously been observed in rat pups [29] and adults [4], also occurs in adolescent rats; (2) systemic pre- or post-retrieval treatment with PROP impaired the reconsolidation of social play-induced CPP; (3) CPP could be reinstated after extinction in vehicle- but not PROP-treated rats; (4) the reconsolidation-window for social reward-related memories is less than 1 h; (5) memory retrieval is necessary for PROP to affect the stability of social reward-related memories; (6) PROP does not affect acquisition, consolidation or retrieval of social reward-related memories. Together, our data show that, concerning the dynamics of social reward-related memories, β-adrenergic neurotransmission specifically mediates the reconsolidation of social play-induced CPP.

In the first experiment, saline-treated animals showed a preference for the social-paired compartment 24 h after post-retrieval treatment, whereas PROP-treated animals did not. This effect of PROP was not the result of a non-specific memory impairment, since PROP treatment in the absence of retrieval did not alter social play-induced CPP [4], [5]. Furthermore, following extinction of CPP, saline-treated animals reinstated their preference 24 h after a reconditioning session, whereas PROP-treated animals did not. Post-retrieval PROP administration has been found to impair memory when animals are re-tested 24 h after retrieval in a variety of paradigms [3,21,22,24-26]. The inability to reinstate the social play-induced CPP response in the PROP-treated group suggests that acute post-retrieval PROP persistently disrupted the social play-CPP memory trace, rather than inducing a retrieval deficit. PROP may have facilitated extinction learning instead of disrupting reconsolidation. However, since extinguished memories can be reinstated after retraining [30], and PROP seems to impair rather than facilitate extinction [31], [32], this explanation is rather unlikely. Somewhat consistent with our results, post-retrieval PROP treatment has previously been shown to disrupt the reconsolidation and reinstatement of cocaine-induced CPP, albeit that a single PROP treatment interfered with reconsolidation, but that repeated post-retrieval PROP treatments were necessary for blockade of reinstatement [22]. In the case of morphine-induced CPP, PROP disrupted reconsolidation but not reinstatement [26]. An important difference between our experiments and these previous studies is the way in which reinstatement was evoked, i.e. a single reconditioning session in the present study vs a drug prime in the previous studies. Another possible explanation for the differences between the abovementioned findings and our results could be that drug reward-context associations might be stronger than natural reward-context associations, so that repeated interference with reconsolidation is necessary to persistently disrupt a drug-induced CPP memory trace [33]. Together, these findings show that β-noradrenergic neurotransmission, involved in reconsolidation of memory for drug [21], [22], [26], [25] and food rewards [24], [25] is also involved in reconsolidation of social reward memories in adolescent rats. Furthermore, PROP persistently disrupted the social-play CPP memory trace as social play-induced CPP could be reinstated in saline- but not PROP-treated animals.

Our results show that the period of instability for social reward-related memories lasted less than 1 h. Using different paradigms, amnesic agents and species, a window of about 6 h after which amnesic treatment no longer affects reconsolidation has often been reported [3,4,34]. Consistent, we found that post-retrieval PROP treatment after a 6 h delay did not impair social play-induced CPP. Interestingly, and in keeping with our findings, two recent studies have shown that amnesic treatments 1 hr post-retrieval do not affect reconsolidation of amphetamine-induced CPP or fear memory [34], [35]. Our data therefore suggest that memory reconsolidation for social play-induced CPP occurs quite quickly. This is not surprising from a mechanistic point of view. Reconsolidation is thought to depend on restabilization of existing synaptic networks [4], and to serve as an updating mechanism for existing memory traces [7]. In this light, a brief reconsolidation-window for social memories may be beneficial for social animals, including humans. Because social animals live in a complex, rapidly changing social environment and social interaction can be very brief, the updating of social information must be rapid in order for social animals to function properly.

Administration of PROP 30 min before the CPP test did not alter the expression of CPP, showing that PROP did not affect retrieval of social reward-related memories. The PROP-treated animals, however, did show an absence of preference 24 h after the test for retrieval, suggesting that, consistent with our first experiment, PROP affected reconsolidation instead of retrieval. Furthermore, in contrast to saline-treated animals, PROP-treated rats did not reinstate their preference for the social-paired compartment. In PROP-treated animals across the different tests in this experiment, the presence and absence of CPP was comparable to that of rats receiving a post-retrieval PROP injection. These findings show that β-noradrenergic neurotransmission is not involved in the retrieval of social reward-related memories, but that blockade of β-adrenoceptors during the retrieval session, and perhaps briefly after, interfered with the reconsolidation of social play-induced CPP. In contrast to our results, PROP has been shown to impair memory retrieval in different paradigms in adult rats and mice [36], [37], but not in humans [38], [39]. Thus, the involvement of β-noradrenergic signaling in memory retrieval likely depends on the type of memory, species and age of the subjects.

Since noradrenergic neurotransmission is known to be involved in acquisition and consolidation of certain types of memories, we tested whether β-adrenoreceptors are involved in the acquisition and consolidation of social play-induced CPP as well. However, daily pre-training or post-training administration of PROP did not affect social play-induced CPP. These results indicate that PROP interferes with synapse-remodeling when the social reward-related memory is reactivated but not when it is formed. Administration of PROP has previously been shown to impair the acquisition of aversive memories in rats and humans [40], [41]. Apparently, involvement of β-adrenoceptors in memory acquisition does not extend to positive emotional memories, although more research is needed to support this suggestion. Unlike memory acquisition, the literature about the effect of PROP on memory consolidation is inconclusive. Post-training administration of PROP has been found to disrupt memory consolidation in some studies [40], [41], but not in others [23], [37], [42], [43]. Again, most of these studies used aversive paradigms to investigate the effect of PROP on memory consolidation, whereas we used an appetitive paradigm. Also, none of these studies used adolescent animals, like the present study. Thus, β-noradrenergic neurotransmission appears to be involved in memory consolidation, but this depends on the type of memory studied and age of the subjects used.

The present study demonstrates that, comparable to adult animals, PROP impairs memory reconsolidation processes in adolescent rats as well. However, unlike the present data, as summarized above, PROP has been shown to disrupt memory acquisition, consolidation [40], [41] or retrieval [36], [37] in adult rats, at least in certain studies. The discrepancies between the role of β-adrenoceptors in these memory processes in adolescent and adult animals may be associated with the age-related changes in noradrenergic innervation of brain structures implicated in learning and memory, such as the hippocampus, amygdala and frontal cortex [44], [45]. Thus, β-adrenoceptor binding has been shown to decline between adolescence and adulthood in cortex [46]. Furthermore, the density of the noradrenaline transporter, likely reflecting noradrenergic innervation, decreases between adolescence and adulthood in frontal cortex and amygdala, but only very modestly so in hippocampus [47], [48]. Although the relationship between noradrenaline transporter and β-adrenoreceptor density during development and their involvement in memory processes is not straightforward, it is not unlikely that some of the discrepancies noted here are the result of developmental changes in noradrenergic innervation. On a more general note, the fact that memory reconsolidation has previously been observed in rat pups [29] and adults [4], may lead to the intuitive assumption that this also occurs in adolescent rats. The present data are, to the best of our knowledge, the first demonstration that this is indeed the case, indicating that memory reconsolidation is a relevant part of memory dynamics throughout the entire lifespan of animals.

Our results demonstrate that in adolescent rats, β-adrenergic neurotransmission mediates the reconsolidation but not the acquisition, consolidation or retrieval of social reward-related memories. This supports the notion that consolidation and reconsolidation of social reward-related memories rely on distinct neural mechanisms. Indeed, several differences in the molecular pathways underlying consolidation and reconsolidation of fear memories have been found [49]–[51]. In keeping with these findings, our results suggest that a distinction between the neural mechanisms of consolidation and reconsolidation also holds for positive emotional memories.

In conclusion, the present study extends our knowledge about memory reconsolidation, showing that social reward-related memories in adolescent rats are subject to reconsolidation after retrieval. In particular, we have demonstrated that treatment with PROP impairs the reconsolidation, but not the acquisition, consolidation and retrieval of social play-induced CPP in adolescent rats. Together, these data show that β-adrenoceptor stimulation is specifically involved in the reconsolidation of social reward memories in adolescent rats. Future studies should determine the neural site of action of β-adrenoceptor-dependent reconsolidation of social play-induced CPP.

## Materials and Methods

### Ethics statement

All experiments were approved by the Animal Ethics Committee of the Utrecht University (license no. 2010.I.04.057) and were in agreement with Dutch laws (Wet op Dierproeven 1996) and European regulations (Guideline 86/609/EEC).

### Animals

Male Wistar rats (Charles River, Sulzfeld, Germany) arrived in our animal facility at 21 days of age and were housed in groups of three or four in 40×26×20 cm (*l*×*w*×*h*) Macrolon cages under controlled conditions (i.e. temperature 20–24°C, 60–65% relative humidity and 12/12 h light cycle with lights on at 7.00 AM). Upon arrival, the animals were allowed at least 5 days of acclimatization to the facility and were handled for 3 days before the start of the experiment. Food and water were available ad libitum. All animals were experimentally naïve and were used only once.

### Apparatus

Place conditioning was performed as previously described [19,20,52]. The place conditioning setup (TSE System, Bad Homburg, Germany) comprised 8 boxes, each consisting of three compartments with removable Plexiglas lids; two equally sized large conditioning compartments (30×25×30 cm; *l* × *w* × *h*) separated by a smaller, neutral compartment (10×25×30 cm; *l*×*w*×*h*). The two conditioning compartments had different visual and tactile cues, which also differed from the cues in the middle compartment. The position of the animal in the apparatus was monitored by an array of photobeam sensors located 2.5 cm above the floor. A computer recorded the time (in msec) the animals spent in each compartment. All experiments with this setup were performed in a sound attenuated and dimly lit room.

### Drugs

(±)-Propranolol HCl (PROP, Sigma-Aldrich, Germany) was dissolved in saline and administered *i.p.* (10 mg/kg, injection volume 2 ml/kg). At doses up to 10 mg/kg, PROP has been shown not to influence social play behavior [53], spontaneous locomotor activity or exploratory behavior [54].

### Statistical analysis

Data were analyzed using SPSS software 15.0 for Windows. For each experiment, the time spent in the social paired and non-social paired compartments were expressed as mean ± SEM. Data were analyzed using ANOVA (mixed-model or two-way, depending on the experiment), using compartment (social or non-social) and treatment (PROP or saline) as between-subjects factor and test-day as repeated-measures factor. ANOVA was followed by Student's paired t-tests when appropriate, to investigate differences between the time spent in the social and non-social compartment.

### Methods

#### 1. Effects of acute post-retrieval PROP on social play-induced CPP

The aim of this experiment was to investigate the effect of an acute post-retrieval PROP injection on the reconsolidation and reinstatement of social play-induced CPP. At 26 days of age (day 1), each rat was placed in the middle compartment of the CPP apparatus and pre-conditioning side preference was determined by allowing the rats to move freely around the three compartments of the apparatus for 15 min (Pretest). On the basis of their Pretest scores, rats were assigned to a compartment in which they would be allowed social interaction during conditioning. We used a counterbalanced place conditioning design [55], meaning that the pre-conditioning preference in each experimental group for rats to be social-paired or non-social paired approximated 50%. Thus, based on their Pretest performance, some rats were conditioned in their preferred compartment, but others were conditioned in their non-preferred compartment. This procedure rules out the possibility that preference shifts are the result of decreased avoidance of the non-preferred compartment. After the Pretest, rats were individually housed to increase their motivation for social interaction and to facilitate the development of social play-induced CPP [19].

Place conditioning began on day 2. Rats underwent eight consecutive days of conditioning, with two conditioning sessions per day. On days 2, 4, 6 and 8 of the experiment, rats were placed for 30 min in one compartment with an initially unfamiliar partner (social session) in the morning, and were placed alone in the other compartment (non-social session) in the afternoon. On days 3, 5, 7 and 9, the order of sessions was reversed, i.e. rats were placed alone in one side of the CPP apparatus during the morning session, and were placed in the other compartment with the social partner in the afternoon session. Social and non-social conditioning-sessions were separated by at least one hour. On day 10, rats were placed in the middle compartment and were allowed to explore the entire apparatus for 15 min (retrieval, RETR), and time spent in each compartment was recorded. Immediately after the retrieval session, the animals were randomly assigned to either the saline- or PROP-treatment group and injected. The next day, the animals were placed in the middle compartment again and were again allowed to move freely in the apparatus for 15 min to investigate the effect of the injection (TEST); this test is also considered the first extinction session. This procedure was repeated once a day for the following days to extinguish place preference, i.e., until the mean difference between the time spent in the social-paired and the non-social-paired compartments was no longer statistically significant for four consecutive days in all the experimental groups. This took between 8 and 22 extinction sessions. Twenty-four hours after the last extinction session, the rats received a reconditioning session. Each rat was placed in the social compartment with a social partner for 30 min (social session) and at least 1 hour later, it was placed in the non-social compartment alone for 30 min (non social session). The next day, the animals were exposed to the whole apparatus for 15 min and preference was determined again (reinstatement, REIN).

#### 2. Effects of delayed post-retrieval PROP on reconsolidation of social play-induced CPP

This experiment was designed to determine the period of instability of the social play-related memory trace after memory retrieval. Animals were conditioned as described in experiment 1. On day 10, one group of animals received PROP or saline 1 h after retrieval while another group of animals received PROP or saline 6 h after memory retrieval. The next day, i.e. 18 h and 23 h after injection, rats were tested (TEST) as described in experiment 1.

#### 3. Effects of PROP on social play-induced CPP in the absence of memory retrieval

This experiment investigated whether memory retrieval is essential for PROP to affect reconsolidation of social play-induced CPP. Animals were conditioned as described in experiment 1. On day 10, instead of a memory retrieval session, animals were treated with PROP or saline in their homecage. The next day, both groups were tested (TEST) as described in experiment 1.

#### 4. Effects of PROP on retrieval of social play-induced CPP

This experiment was designed to investigate the effect of PROP on retrieval of memory for social play-induced CPP. Animals were conditioned as described in experiment 1. PROP or saline was injected 30 min before the memory retrieval session. Animals were tested for reconsolidation (TEST) and reinstatement (REIN) as described in experiment 1.

#### 5. Effects of PROP on acquisition and consolidation of social play-induced CPP

These experiments investigated the effects of PROP on acquisition and consolidation of social play-induced CPP. Animals were conditioned as described in experiment 1. Thirty minutes before or immediately after each conditioning session, animals were treated with PROP or saline, to investigate the effect of PROP on acquisition and consolidation of social play-induced CPP, respectively. On day 10, the animals were tested as described in experiment 1 (TEST).
