# Correction: β-Adrenoreceptor Stimulation Mediates Reconsolidation of Social Reward-Related Memories

**DOI:** 10.1371/annotation/5c641529-246f-43c7-a86a-ef2c0488dbc1

**Published:** 2013-09-23

**Authors:** E. J. Marijke Achterberg, Viviana Trezza, Louk J. M. J. Vanderschuren

Three references were omitted from the PDF during the preparation of this article for publication.

These references are:

53. Vanderschuren LJMJ, Trezza V, Griffioen-Roose S, Schiepers OJG, Van Leeuwen N, et al. (2008) Methylphenidate disrupts social play behavior in adolescent rats. Neuropsychopharmacology 33: 2946–2956. doi: 10.1038/npp.2008.10.

54. Sara SJ, Dyon-Laurent C, Hervé A (1995) Novelty seeking behavior in the rat is dependent upon the integrity of the noradrenergic system. Cogn Brain Res 2: 181–187.

55. Tzschentke TM (2007) Measuring reward with the conditioned place preference paradigm: update of the last decade. Addict Biol 12: 227–462. 

